# Adapting a complex violence prevention intervention: a case study of the Good School Toolkit in Uganda

**DOI:** 10.1186/s12889-024-17676-x

**Published:** 2024-02-09

**Authors:** Heidi Grundlingh, Nambusi Kyegombe, Sophie Namy, Janet Nakuti, Yvonne Laruni, Barbrah Nanyunja, Hassan Muluusi, Mastula Nakiboneka, Aggrey Mukuwa, Clare Tanton, Louise Knight, Dipak Naker, Karen Devries

**Affiliations:** 1 Child Protection Research Group, Department of Population Health, London, School of Hygiene and Tropical Medicine, London, UK; 2 Raising Voices, Kampala, Uganda

**Keywords:** Adaptation, Multicomponent, Complex intervention, School, Violence prevention

## Abstract

**Background:**

Adaptation is a key strategy to extend the reach of evidence-based interventions to prevent violence in new populations, but there is a dearth of practical case examples. The Good School Toolkit was developed by Ugandan NGO Raising Voices for use in primary schools (GST-P). We describe our systematic approach to adapting the GST-P for use in secondary schools in Uganda, and reflect on the utility of the process as well as limitations of existing adaptation frameworks.

**Methods:**

We adapted the GST-P in four phases, which included: I) clarifying the logic model and core intervention components using a streamlined process; II) conducting formative research (cross-sectional survey, focus groups, etc.) to understand the new population; III) selecting and preparing new intervention components and modifying existing intervention components; and IV) pretesting new intervention components with teachers and students in Uganda.

**Results:**

We identified core components using a logic model. Formative research showed results largely in line with our apriori hypotheses. Teacher violence remained highly prevalent in secondary versus primary schools (> 65% of secondary students reported past year exposure), while peer violence significantly increased (secondary = 52% vs. primary girls = 40%, *P* < 0.001; secondary = 54% vs. primary boys = 44%, *P* = 0.009) in secondary versus primary schools. Significantly more secondary girls (51%) than secondary boys (45%) reported past year dating/intimate partner violence (*P* = 0.03). Inequitable, gendered educational practices emerged as a salient theme, perceived to heighten female students’ vulnerability to violence. In light of these findings, we made several adjustments to the adapted intervention. We strengthened existing teacher and peer violence intervention components. We also developed, pretested and revised new program components to prevent dating violence and promote ‘gender fairness in schools’. Finally, original activities were modified to support engagement with school administration and promote increased student agency in secondary schools.

**Conclusions:**

Based on our experience, it was difficult to apply mechanistic models to clarify the intervention logic of the GST-P, a complex multicomponent intervention, and simpler methods may be sufficient. Our team had high levels of contextual knowledge before the adaptation, and formative research to understand the new target population provided only limited additional insight. In similar situations, a simplified approach to mapping the core intervention components, qualitative research to understand the new target population, and pre-testing of new intervention components may be the most informative elements of systematic adaptation processes.

**Supplementary Information:**

The online version contains supplementary material available at 10.1186/s12889-024-17676-x.

## Introduction

It is estimated that 1 billion children globally have experienced emotional, physical or sexual violence in the past year [1], and school is a prime risk environment where children and adolescents are exposed to such violence [2]. The recently published national survey of Violence Against Children in Uganda found that 59% of young females and 68% of young males reported experience of physical violence, 34% young females and 36% of young males reported emotional violence, and 35% of females and 17% of males reported sexual violence under age 18. More than 1 in 10 girls and 1 in 20 boys experienced all three types of violence in childhood. Common perpetrators include teachers, peers and intimate friends, all of whom interact with students in school settings [3].

Longitudinal data show that experience of violence in childhood is associated with negative health and social outcomes including depression [4]; sexual risk behaviours [5]; poor educational outcomes [6]; and development of conduct disorder [7], which predicts later use of violence in adult relationships [8]. Research into prevention and response to violence against children in schools has been outlined as a priority [9, 10]. Despite the high prevalence of violence against children at schools, few interventions exist to prevent multiple forms of school violence in low and middle income countries and even fewer have been rigorously evaluated [11, 12]. Exceptions include the Good School Toolkit [13, 14] in Uganda and the 130 session Right to Play Intervention, trialled in Grade 6 students in Pakistan [15]. The paucity of effective interventions underscores the need for both additional intervention development and testing, and research on adaptation of existing highly successful interventions. Both are necessary to address the huge unmet need for violence prevention in schools.

### The Good School Toolkit for primary schools

The Good School Toolkit for primary schools (GST-P), developed by Ugandan NGO Raising Voices (www.raisingvoices.org), reduced the risk of physical violence by 42% from school staff towards primary school students aged 11–14 and also reduced staff emotional violence, and peer physical and emotional violence [13, 14]. GST-P takes a whole-school approach to prevent violence against children and contains more than 60 different activities and core structures that schools can choose to implement. The GST-P is implemented over 18 months, and is targeted at 4 entry points believed to create a non-violent and positive operational culture: respectful student–teacher relationships, student voice and participation, transparent school administration, and engagement from student caregivers [16]. It draws on the Transtheoretical Model [17] by framing school-level change as a 6-step process. It addresses power dynamics in relationships, which are thought to underpin multiple forms of violence behaviour. Materials include books describing activities and booklets, posters, tools and example documents used to reinforce key ideas. The GST-P is publicly available at www.raisingvoices.org. The toolkit is implemented by two teacher and two student protagonists in each school with support from Raising Voices or another local child protection/child rights NGO.

Given the effectiveness of the GST-P in reducing both teacher and peer violence we sought to adapt the GST-P to tailor the intervention for use in secondary schools in Uganda. We hypothesised adaptation would be needed to reflect the different patterns of violence from staff and peers in secondary schools compared to primary schools, the changing developmental levels of students as they transition from early to late adolescence, and the different context and operational culture in secondary schools.

### Current guidelines and methods for adapting evidence-based interventions

Identifying and adapting evidence-based violence prevention interventions for new populations is a ‘critical strategy …to accelerate efforts’ to prevent violence and other social and health disparities [18]. However, adaptations of interventions to accommodate differences in new populations are often ad-hoc and may unintentionally dilute programme effectiveness [19, 20]. There are several step-by-step adaptation guidelines which outline the systematic adaptation of multicomponent interventions so as to maintain fidelity and intervention effectiveness [18, 20–22], with adaptation described as ‘the process of modifying activities and delivery methods of an evidence-based intervention, without contradicting the core elements, theory, and internal logic thought most likely to produce the intervention’s main effects’ [20]. While these guidelines may inform adaptation processes, relatively few published empirical studies describe case studies of adaptation in enough detail to replicate for other populations [20, 23, 24].

In this article we present a case study using a systematic approach for adaptation of the Good School Toolkit for primary to secondary schools in Uganda. The adaptation focused primarily on content, rather than delivery model. We provide a description of our adaptation methods, and show how findings informed the adaptation of intervention components. Finally, we discuss our reflections on the utility of different aspects of the adaptation process and discuss implications for the adaptation field.

## Methods

**Fig. 1:**
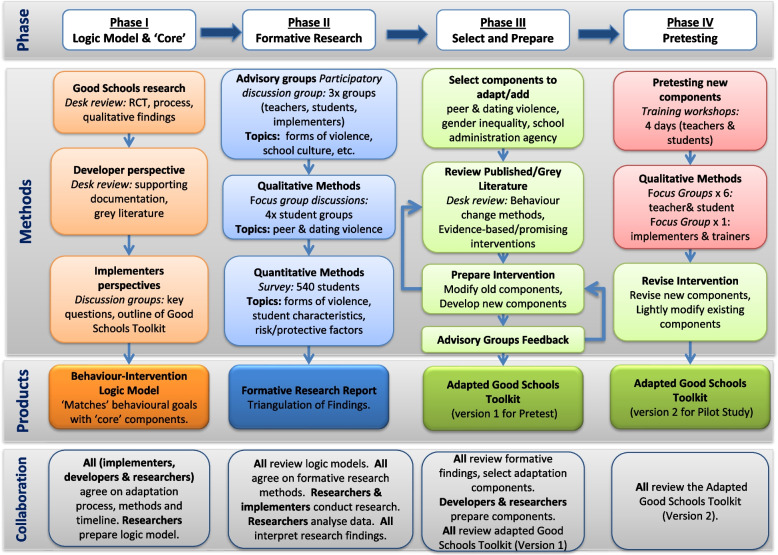
Adaptation of the Good Schools Toolkit for Secondary Schools

Drawing on guidance from the Centers for Disease Control [20] and others [18, 22], we adapted the GST-P in four phases, which involved: I) clarifying the intervention logic model and core components; II) conducting formative research to understand the new population; III) selecting and preparing new intervention components and modifying existing intervention components; and IV) pretesting new intervention components with the new population (see Fig. 1).

The adaptation was part of an existing collaboration between Raising Voices (which developed and implemented the GST-P) and researchers from the London School of Hygiene and Tropical Medicine (LSHTM). The LSHTM research manager (HG) was based in Uganda and worked closely with Raising Voices staff. We established an LSHTM/Raising Voices steering committee (formed of the director and staff from Raising Voices and the PI and research staff from LSHTM) which held quarterly review meetings to discuss emerging research findings to support Raising Voices’ decision making about the content of the adaptation.

### Ethics approval and consent to participate

For data collection activities with research participants, we utilised a 3-tiered consent strategy (48). Consent for school participation in the study was sought from head teachers; parents of students were informed about the research and were able to opt students out of participation in the research (passive consent); and students themselves provided informed consent. All children who participated in data collection activities were offered counselling, and all of those who disclosed experience of abuse were referred to an independent child protection partner, which then facilitated connections with health, social and legal services as appropriate. Approval for all procedures, including passive consent, was provided by the ethics committees of the London School of Hygiene and Tropical Medicine, MildMay Uganda and the Uganda National Council for Science and Technology (UNCST). All data collection was carried out in accordance with relevant guidelines and regulations.

### Adaptation process & methods

#### Phase I. Clarifying the logic model and core components of the GST-P

Logic models link intervention goals and related behaviours with specific intervention components or activities [18]. There are various established techniques [18, 25] to create logic models, which involve identifying behaviours which interventionists aim to change, the determinants of those behaviours, and intervention activities which will act to address the determinants of those behaviours.

We found that identifying determinants for our large and complex intervention unwieldy in practice and therefore developed a succinct ‘Behaviour-Intervention Logic Model’ for the original intervention (GST-P). This model involved less emphasis on detailed mapping of determinants, which were frequently overlapping across behaviours. We extracted intervention goals, related behaviours, and intervention components by drawing on Raising Voices’ intervention materials and theory. Intervention components were then identified as ‘core’ (central to effective implementation) and linked to specific related behaviours if they were used or frequently used and were perceived to be effective in addressing that related behaviour. We identified the core components based on previous research [13, 26, 27], Raising Voices’ theory of change, and the perspectives of programme staff based on their experience implementing the GST-P. In the GST-P, core components could be workshops, activities, or other sessions.

#### Phase II. Formative research to understand the new population

The aim of the formative research was to understand the context of secondary schools as compared to primary schools, by exploring the prevalence of different forms of violence, gender and relationship power, and relationship dynamics. We purposively selected one rural and one urban school, from a list of secondary schools with > 500 pupils, with no previous exposure to the intervention, and which were typical of either a rural or urban school based on the judgement of Raising Voices’ staff (that is, not especially well or under-resourced, not an outlier in terms of academic performance). We conducted focus group discussions (FGDs) and a cross-sectional survey in both schools.

##### Focus group discussions

The aim of the FGDs was to explore student experiences of peer violence, adolescent dating relationships and intimate partner violence. Male and female students were purposively sampled from both schools based on their age and representing the following groups: student leaders, popular and less popular students, and students who were regularly reprimanded for poor behaviour. These groups were selected to represent key student positions in the Toolkit intervention, and also students who may engage less with the Toolkit. The different groupings were identified in a pre-focus group workshop with students and teachers. The student counsellor from each school was asked to identify students from each of these groupings. Students were excluded if they were non-Luganda speakers. We conducted four FGDs (two male and two female groups), of between 8 and 12 participants. FGDs were audio-recorded.

##### Cross sectional survey

The aim of the survey was to determine the characteristics of secondary school students, whether the prevalence of teacher and peer violence differed between primary and secondary school populations and levels of dating/intimate partner violence among secondary school students. We selected interviewers who had extensive experience with violence research. They underwent two weeks of training to reliably administer survey instruments and to observe ethical and referral protocols. In both secondary schools, a list of all students was obtained. We obtained a simple random sample of 560 students across all grades. Sampled students were then approached by interviewers, provided with verbal and written information about the study and asked if they would like to consent to participation. Students were excluded if they did not speak Luganda. 497 students completed surveys and 63 students did not. Reasons for non-completion were because they had left the school (*n* = 29), did not speak Luganda (*n* = 22), were absent (*n* = 7), refused (*n* = 3) or there was an error in the class list (*n* = 2).

Data were collected by interviewers on tablet computers and individual survey interviews took place in private locations, where responses could not be overheard. Students' experiences of violence from teachers, peers and others were assessed using the ISPCAN Child Abuse Screening Tool—Children's Institutional Version (ICAST-CI) [28] and other measures. A description of all measurement instruments and demographic characteristics of student respondents is available in Additional file 1, Tables S1a and S1b.

#### Phase III. Selecting and preparing new intervention components and modifying existing intervention components

To select intervention components, the Raising Voices/LSHTM steering committee considered previous research findings [13, 14, 27], the GST-P core components and the behaviours these aim to address identified in Phase 1, and the types of violence and salient themes identified in secondary schools during Phase 2. Upon steering committee consensus that the intervention would be a ‘good fit’ for the new population, we identified intervention gaps and selected new program content for development or existing components for modification [20].

To prepare new components, we outlined specific component objectives based on the behavioural determinants (knowledge, attitude, skills, perceived social norms) linked to behaviours we sought to change. We identified evidence-based interventions which address adolescent dating violence by searching academic literature for systematic reviews of interventions [11, 12, 29, 30]. See Additional file 2, for the ‘Review methodology and summary findings’. In a few instances, content from these evidence-based interventions or other promising interventions were used to guide content development, with permission from the authors [31–34]. Mostly, original content was developed through a collaborative and iterative process which drew on the contextual knowledge of the developers, implementers and advisory groups, and the formative research findings.

#### Phase IV. Pretesting new intervention components

We tested all new intervention components in small groups of teachers and students, under conditions that mimicked normal implementation. These groups were recruited via the rural school selected in the formative research (Phase II) and conducted at a conference facility nearby. Twenty three of the 52 teachers employed by the rural school were available to attend the teacher workshops and all 23 were invited; 17 of these attended the workshops all four days. Students were purposively selected to represent different age and peer groups. Eighteen male and 18 female students from different academic years were invited to attend the student workshops; at least 15 male and 13 female students attended all four days. The workshops were delivered by Raising Voices staff. To understand participant perspectives, we conducted six FGDs with teacher and student workshop attendees, purposively sampled based on their ability to articulate nuanced positions during the workshops and attendance at all four days of workshops; no individuals who were invited to participate declined. Separate teacher, male student and female student focus groups each included 8 to 9 participants who provided informed consent. Similar data collection methods were used as described in Phase II.

### Data analysis

#### FGDs (phases II and IV)

FGDs were translated and transcribed for analysis. For the formative research (Phase II), we prepared detailed notes organised by topic based on a priori lines of inquiry. This analytical approach has previously been used in adaptation work [19]. For the pretesting (Phase IV), analysis was thematic, using a framework approach [35], with exploration of participant comprehension, acceptability and applicability. During analysis, detailed analytical memos were prepared on each theme, including recommendations for revisions to the intervention components.

#### Survey (phase II)

We used Stata 13 for all data analyses [36] accounting for clustering at the school level. We performed a descriptive analysis for all participating secondary school students (*N* = 497) and assessed the prevalence of dating/intimate partner violence for those who had ever had an intimate partner (*N* = 268). We compared past year prevalence of teacher and peer violence reported by secondary schools students (*N* = 393, excluding first year students) with our primary school data (*N* = 3706) [13]. Comparisons were done using the chi-squared test, chi-squared test for trend for categorical variables or the t-test for continuous variables.

#### Integration of findings

The qualitative and quantitative findings from the formative work (phase II) were integrated via discussion. We presented and discussed findings and sought to triangulate findings from different methods and understand where they converged; where each method was adding additional complementary and/or contrasting findings, and to build from these insights to expand the range of insights into how to GST should be adapted [37].

### Stakeholder engagement

We convened three groups of ‘users’ during phase II of the adaptation. These groups were conceptualised as collaborators in the development of new intervention content, rather than as participants in research. They include a teacher group of 12 teachers, a student group which included 14 secondary students; both groups represented several schools. We also convened a third group of 8 programme implementers, across several organisations. Groups were mixed by gender and age. Each of the groups met three times over a 12-month period. At group meetings, we used participatory learning and action methods [38] to explore perspectives on the nature of violence from teachers and student peers, gendered educational practices in schools, and other topics which emerged as important. We created feedback loops throughout the adaptation process to validate the findings from the focus group discussions and filter some of the ideas for new components with the advisory groups. The meetings were recorded, transcribed, and extensive notes were prepared.

## Results

### Phase I. Logic model and core components of good school toolkit for primary schools

We created a Behaviour-Intervention Logic Model of the GST-P, indicating ‘core’ and ‘not core’ components. An extract of this is presented in Table 1. For example, the related behaviour ‘Teachers foster … participation in positive discipline…’ was linked to Step 4, Activity 4.6 (Students’ Court) of the GST-P based on student reports of considerable exposure to the Students’ Court proceedings [39] and implementers’ view that this intervention component was highly effective in facilitating student participation in positive discipline. If activities were not frequently used and could not be linked to a related behaviour, they were considered ‘not core’ and could be discarded if needed in adaptation.

**Table 1:** Extract of the Behaviour-Intervention Logic Model for the Good School Toolkit for primary school

**Behaviour-Intervention Logic Model**		
**Intervention Goals**	**Related Behaviours**	**Intervention Components (Workshops or Activities)**
Teachers use positive discipline methods to facilitate positive self-discipline in students	• Teachers rethink/embrace their role as teacher. (matched to GST Step 3.1, 3.2, 3.3, 3.4, 3.6) • Teachers practice positive discipline methods and zero corporal punishment with individual students, in the classroom and in the school (matched to GST Step 4.1, 4.2, 4.3, 4.5, 4.6, 4.7) • Teachers foster gender equity, and student voice and participation in positive discipline and decisions which affect them. (matched to GST Step 3.3, 4.4, 4.5, 4.6, 4.7)	Good School Toolkit Step 3 Activities: **3.1 Create an Implementation Plan** **3.2 Leadership Workshop 3: School Staff** 3.3 **Student–Teacher Relationships** (E.g. Assembly to facilitate teacher-student dialogue.) 3.4 Creative Teaching 3.6 Professional Goals & Feedback Good School Toolkit Step 4 Activities: 4.**1 Create an Implementation Plan** 4.2 **Leadership Workshop 4: School Staff** 4.3 Reinforce Positive Discipline Commitment by school staff 4.4 **Recognize Student Strengths** (E.g. Student Wall of Fame) 4.5 **Setting Classroom Rules** 4.6 **Students’ Court** (Proceedings led by students to decide on minor student disciplinary cases.) 4.7 School Standards and Rules (Developed via whole-school participation.)

‘Core’ intervention components are in bold text (more frequently used and linked to a related behaviour) and in plain text (moderately used and linked). Components perceived as ‘not core’ (not frequently used or not closely linked) are in strikethrough text

### Phase II. Formative research

#### Teacher violence

We hypothesized that emotional and physical violence from teachers would be less prevalent in secondary schools than primary schools, while sexual violence would increase. However, we found that secondary girls and boys reported significantly more past year emotional violence from school staff than primary students (girls: 33.7% vs. 26.0%, *P* = 0.02; boys: 35.0% vs. 26.2%, *P* = 0.008; secondary vs. primary; Table 2). Past year physical violence from staff against secondary students remained highly prevalent, although secondary girls experienced less physical violence compared to primary girls (60.2% vs. 70.3%, *P* < 0.001, secondary vs. primary; Table 2). Secondary girls reported significantly more past year sexual violence from school staff compared to primary girls (secondary = 5.6% vs. primary girls = 2.1%, *P* < 0.001; Table 2). These findings were consistent with qualitative data collected during the focus group discussions and from the student advisory group. Students shared that secondary teachers often used corporal punishment (‘beating’) to punish misbehaviour and poor academic performance, and discourage dating:

*“When the teachers implicate you for coupling [dating] …they beat you at the assembly…” (female student, focus group discussion).*

**Table 2:** Prevalence of violence reported by male and female students in primary and secondary schools

	**Male Students**					**Female Students**				
**Prevalence of Violence Reported by Students**	**Primary School**		**Secondary School**		***P***^**a**^	**Primary School**		**Secondary School**		***P***^**a**^
	**(***N*** = 1769)**		**(***N*** = 197) ~**			**(***N*** = 1937)**		**(***N*** = 196) ~ **		
**From School Staff (Past Year)**^**b**^										
Emotional Violence	464	26%	69	35%	0.008	504	26%	66	34%	0.02
Physical violence	1,108	63%	134	68%	0.25	1,362	70%	118	60%	< 0.001
Severe^c^ physical violence	98	6%	14	7%	0.18	124	6%	17	9%	0.11
Sexual Violence	26	2%	4	2%	0.3	40	2%	11	6%	< 0.001
***Any emotional, physical, sexual violence***	***1180***	***67%***	***146***	***74%***	***0.01***	***1414***	***73%***	***134***	***68%***	***0.11***
**From Student Peers (Past Year)**^**b**^										
Emotional Violence	584	33%	105	44%	0.006	517	27%	89	38%	< 0.001
Physical violence	442	25%	73	29%	0.3	438	23%	57	22%	0.67
Sexual Violence	28	2%	15	8%	< 0.001	75	4%	43	20%	< 0.001
***Any emotional, physical, sexual violence***	***779***	***44%***	***131***	***54%***	***0.009***	***769***	***40%***	***125***	***52%***	**< ***0.001*
**From dating or intimate partners (Lifetime)**^**d**^			**Secondary School Male**					**Secondary School Female**		*P*^***e***^
			**(***N*** = 132)**^**d**^					**(***N*** = 136)**^**d**^		
Emotional Violence			*52*	39%				*60*	44%	0.43
Physical Violence			*2*	2%				*6*	4%	0.16
Sexual Violence			*12*	9%				*27*	20%	0.01
***Any emotional, physical, sexual violence***			***55***	***42%***				***70***	***51%***	***0.03***

^a^*P*-value for comparison between secondary and primary school students

^b^For 'past year' violence from school staff (male *N* = 197, female *N* = 196); first year students excluded as they did not attend secondary school in the preceding year

^c^Cut you with a sharp object, burnt you, choked you or forced you to do something that was dangerous or whipped you; severely beaten you up

^d^Prevalence of violence analysed for those who had ever had a dating or intimate partner *N* = 268

^e^*P*-value for comparison between male and female students

Some students perceived that secondary girls may be beaten less but received verbal insults instead.

#### Peer violence 

We hypothesised that sexual violence from peers would be more common in secondary versus primary schools, while emotional and physical violence would be less common. Past year sexual violence from secondary school peers was significantly more common, compared to primary students (girls: 20% vs. 4%, *P* < 0.001; boys: 8% vs. 2%, *P* = 0.001; secondary vs. primary; Table 2). Contrary to our hypothesis, secondary students also reported significantly more emotional violence (girls: 38% vs. 27%, *P* < 0.001; boys: 44% vs. 33%, *P* = 0.006; secondary vs. primary; Table 2), while physical violence from peers remained prevalent in secondary school (reported by 22% of girls and 29% of boys). A recurring theme around peer violence was the desire to feel powerful, for example, boys ‘sabotaging a bright girl’ because she performs better than boys, and the abuse of power by student leaders when ‘beating them [students] for no reason’ or ‘ordering others [student-peers] to do things’. One student related peer violence to situations where peers attempt to assert power over others (students used ‘bullying’ loosely to describe isolated instances and widespread peer violence):

*“I understand bullying [isolated instances and widespread peer violence] as being despised, someone despises (you) because they think that at that moment they are better than you, … they [perpetrators] feel that they are better than you and they want you to do all the work for them.”* (male student, focus group discussion)

#### Adolescent dating and intimate partner violence

More than 50% of all secondary students surveyed reported ever being in a dating or intimate relationship. The lifetime prevalence of dating/intimate partner violence was analysed for all students who had ‘ever partnered’ (males *N* = 132, females *N* = 136). In line with our hypothesis, secondary girls reported significantly higher levels of sexual violence from dating partners (secondary girls = 20% vs. secondary boys = 9%, *P* < 0. 01, see Table 2), which typically included acts of sexual touching (14%), sexual coercion (7%) and forced sex (7%) (not presented). In FGDs, boys reported that girls are coerced by boys/males who repeatedly pressure them for sex. Girls feared that their partners will end the relationship, or girls felt obliged to perform sexual favours due to receiving gifts from their partners. Some boys also reported male attitudes which condone forced or coerced sex:

*“Boys have a mentality that a girl’s ‘no’ is a ‘yes’. And that is the case in most cases. In most cases girls will say ‘no’ even when they want it.”* (male student, focus group discussion)

NGO staff, teachers and students indicated that adolescent dating/intimate relationships are strongly prohibited by parents and teachers. Adolescents typically do not openly engage in dating relationships and may have under reported their dating practices. The advisory groups cautioned that teachers and school administration may have reservations about openly addressing adolescent dating violence at school, lest it be seen as condoning dating relationships. Increasing levels of sexual violence from teachers, peers and dating partners towards girls emerged as a recurring theme, and thus priority area for intervention adaptation.

#### Gender inequality and the gendered nature of school violence

Planned subgroup analysis of our GST-P trial data showed that the intervention was highly successful at reducing past week physical violence from school staff, but that the effect was stronger in boys (OR = 0.34, 95%CI 0.21–0.56), than girls (OR = 0.46, 95%CI 0.29–0.74), p value for interaction 0.0431 [14]. We explored qualitatively how gendered practices in secondary schools may have placed girls at risk for violence and hindered intervention effects in girls. Secondary school students reported that teachers prefer boys for school leadership roles, as:

*‘they [teachers] still hold traditional beliefs that a man has to be higher than a woman, they [women] have to be below [men]’* (female student, student user group)

Male students suggested that a woman should not hold a more senior career or leadership position than her husband as she may not respect him in married life:

*“Women should get to know that a man is always above her. That is total respect. She should care about the husband, be kind, and show total respect…They should kneel down for their husband [referring to a cultural practice at ceremonies and weddings].”* (male student, student user group)

Gender imbalances in educational practices and opportunities for girls, upheld by inequitable gender norms which are internalised by teachers and students alike, may hinder girls’ participation and benefit from intervention activities. Female and male students’ acceptance of male superiority and entitlement may also increase female students’ vulnerability to violence. The gendered nature of teacher, peer and dating/intimate partner violence was identified as a key emerging theme in our formative research, not explicitly addressed in the original intervention, hence requiring the addition and modification of intervention components.

#### Agency and ownership in secondary schools

Teacher advisory user groups indicated that educators were under immense pressure by parents to ensure academic performance in secondary school students. This resulted in extended hours of teaching and increased use of corporal punishment for poor academic performance. The teacher advisory group perceived that secondary schools had more dynamic operational management and higher levels of professional training of teaching staff, relative to primary schools. The study team thus hypothesised that secondary school administrations’ agency and ownership could be fostered by providing the school administration with a more defined role and specific school-based tasks, relative to the primary school intervention. Similarly, adolescent students have emerging reasoning, organisational and relational capacities [40], in a way that is different to primary school students. Hence, the study team also hypothesized that it would be beneficial to adapting and/or add content to the intervention to provide secondary school students with more opportunities for ownership and agency. For example, we adapted the content to equip adolescent students to run school-wide campaigns to prevent peer violence, dating violence and gender imbalances in their schools.

### Phase III. Selection and preparation of intervention components

The LSHTM/Raising Voices steering committee selected specific intervention components to modify or add in order to address: 1. teacher and peer violence, 2. adolescent dating violence, 3. gender inequality in schools, and 4. school administration and student agency and ownership of the intervention. Sexual violence towards girls was a recurring theme addressed under each of these salient themes, but also by the addition of a cross cutting component on the use/misuse of power in relationships. These modified or additional components were mapped onto the Behaviour-Intervention Logic Model. Hence, components were integrated while maintaining the ‘core components’.

We iteratively prepared content for the components. See Table 3 for the content outline of the adapted Good School Toolkit-Secondary (GST-S). New components (activities and workshops) were prepared in line with agreed component objectives and based on behavioural determinants relevant to the context. For example, the objectives for the Workshops 2.4 and 2.5 on gender included empathizing with unfairness toward girls at school (addressing attitudes/beliefs), and planning how to treat girls more equally at school (building skills and self-efficacy, preparing for change). Based on the objectives for each new component, we collaboratively outlined drafts of new workshops or activities. The curriculum writer then developed the sessions in detail, while the researcher checked that the new or modified components appropriately applied evidence-based behaviour change methods to target the specified behavioural determinants [41, 42].

**Table 3:** Overview of the adapted ‘Good School Toolkit -Secondary’ content

**Step**	**School-led Activities**	**Content of Leadership Workshop Modules**
**STEP 1—Creating Team/Network**	1.1 Good School Network 1.2 Admin* Introduces GST to School **1.3 Recruit Teachers to GSC** **1.4 Recruit Students to GSC** **1.5 Recruit Community Members to GSC** 1.6 Recruit Admin* to GSC **1.7 Subcommittee Welcome Meetings** **1.8 Leadership Workshop 1: GSC Training (WS 1.1–1.8)** 1.9 Good School Morning 1: Our Shared Rights (WS 1.6)	**1.1 What Is a Good School?** 1.2 Creating a Conducive Learning Environment 1.3 What Is a Good Teacher? 1.4 Creating Positive Discipline at Your School **1.5 What Is Good Governance?** 1.6 Our Share Rights 1.7 Four Types of Leaders **1.8 Using Participatory Facilitation**
**STEP 2—Preparing for Change**	2.1 Create Plan **2.2 Survey** 2.3 Bulletin Board **2.4 Leadership Workshop 2: GSC Training (WS 2.1–2.7)** **2.5 School-Wide Initiatives and Activities** 2.6 Good School Morning 2: Four Types of Leaders (WS 1.7) 2.7 One-Week Power Campaign 2.8 Launch GST	2.1 Types of Power **2.2 Types of Violence** 2.3 Peer Violence 2.4 Gender in Schools **2.5 Challenging Gender Roles** **2.6 Sexual Violence in Schools** **2.7 Revisiting Participatory Facilitation**
**STEP 3—Good Teachers/Teaching**	3.1 Create Plan **3.2 Leadership Workshop 3: School Staff (WS 3.1–3.8)** **3.3 Student–Teacher Relationships** **3.4 Creative Teaching** **3.6 Professional Goals & Feedback** 3.7 Good School Morning 3: Gender in Schools (WS 2.4) 3.8 Gender Campaign	**3.1 Remembering Relationships** 3.2 Professional Pride 3.3 Challenging Gender Roles 3.4 Teaching for Both Genders **3.5 Creating Teaching Techniques** 3.6 Why Do Students Misbehave? 3.7 Being a Role Model 3.8 Why Go to a Good School? (Peer Pressure)
**STEP 4—Positive Discipline**	4.1 Create Plan 4.2 Leadership Workshop 4: School Staff (WS 4.1–4.7) 4.3 Reinforce Positive Discipline Commitment 4.4 Recognize Student Strengths 4.5 Classroom Rules 4.6 Student Court **4.7 School Standards and Rules** 4.8 Good School Morning 4: Peer Violence (WS 2.3) 4.9 Peer Violence Campaign	4.1 What Is Corporal Punishment? 4.2 Corporal Punishment on Trial 4.3 Punishment vs. Discipline 4.4 Why Voice Matters 4.5 Positive Discipline Responses 4.6 Positive Discipline Role-Role Play 4.7 Encouraging Good Behavior
**STEP 5—Good Learning Environ-mint**	5.1 Create Plan **5.2 Create Code of Conduct** 5.3 Share Code of Conduct 5.4 Student Leadership Opportunities 5.5 Prepare Students for Leadership (peer to peer) 5.6 Create a Student Referral Directory 5.7 Engage the community in caring for the Physical Compound 5.8 Good School Morning 5: Sexual Violence in Schools (WS 2.6) 5.9 Good School Parent’s Day	
**STEP 6—School Governance/ Way Forward**	6.1 Create Plan 6.2 Good School Morning 6: Why go to a Good School? (WS 3.8) 6.3 Good School Assessment 6.4 Defining Way the Forward 6.5 Transition Meeting 6.6 Community Celebration	

The original or unchanged Good School Toolkit content is in plain text, deleted content. the strengthened content in **bold text** and the new content underlined. GSC is Good School Committee, GST-Good School Toolkit, Admin-school administration, WS-Workshop

### Phase IV. Pretesting intervention components

Full pretest findings and recommended intervention revisions are in Additional file 3. Here we present the pretest findings for two workshops which address gender (see Table 3, Workshop Modules 2.4 and 2.5), and briefly describe how the workshops and intervention were revised. Workshop 2.4, ‘Gender in schools’ was designed to generate empathy and insight as teachers and students listen to the story of a secondary school girl who encounters social and educational challenges because she is a girl. The second workshop, originally called ‘Challenging gender roles’ (see Table 3, Workshop Module 2.5) explored how gender roles are socially constructed and can change over time, and the benefits of gender equality in education. Analysis of the FGDs indicated mixed views on the acceptability from teachers on content about gender balanced leadership opportunities and academic support. Some teachers reported concerns that gender equitable socialisation at school would erode future patriarchal structures in family life:

*“I cannot imagine having a wife in my house that has the same education… giving them[girls] the same opportunities are putting them at a disadvantage after school*.” (male teacher).

Students also showed mixed acceptability towards gender balanced practices at school, with the majority of male students not supportive of equal treatment for girls in school; that is, in terms of leadership and equal gender roles in peer relationships. They are mostly concerned that girls ‘…will assume equal power once they grow up to have families…’ (male student) which will threaten existing patriarchal practices of female submission in marriage. Boys would like to ‘maintain their manhood’ (female teacher); that is, their masculine identities based on the idea that males are superior. Girls internalised examples of gender equality and expressed their intention to have agency in future situations of gender imbalance:

*“My thoughts have changed [since attending the workshops], because previously I was thinking that it is the boy who has the power, but now I know that I also have power, … now we know that no one is above the other, we all have to be equal.”*(female student)

Hence, feedback from teachers and students indicated that it is paramount to promote equal educational and leadership opportunities for girls, but we decided to refocus the content on ‘gender fairness’ for both boys and girls in school. Encouraging reflections on fairness was perceived as more palatable rather than overtly challenging societal gender roles, as the latter may have alienated male participants. For example, we changed the original name of Workshop Module 2.5 from ‘Challenging Gender Roles’ to ‘Gender Fairness in Schools’. We also restructured the workshops to primarily focus on fair treatment and equal opportunities for both girls and boys in schools based on mutual understanding and respect, and the benefits of equal educational opportunities for both sexes and society. In addition, user-friendly pamphlets for teachers and students were developed to introduce a new way of talking about gender through an analysis of power in interpersonal relationships.

## Discussion

### Summary of key findings

We retained much of the Toolkit and strengthened the content around teacher and peer violence. The main areas which emerged for which new content needed to be developed for secondary school populations were around gender inequity and patriarchal norms, adolescent dating violence and sexual violence against girls, and to support the increasing agency of students. We also found that new content around how to engage with busy school administrations would be important. We found however that overtly challenging patriarchal gender norms was not likely to be viewed as acceptable by intervention participants, and revised these new components to provide less overt challenge while still including much of the same content.

For our adaptation process, we were able to draw on existing guidance, but had to make several adjustments and streamline processes in order to actually achieve an adapted intervention prototype. We found that existing tools to map intervention logic models and components were too unwieldy to use in practice with our complex, multicomponent intervention, so we streamlined several of these. We did find that the pre-existing contextual knowledge within the Raising Voices team, as well as our qualitative research and pre-testing of new components, were essential in understanding some of the potential relational and systems dynamics likely to affect implementation in our new target population, including gender inequity and school administration priorities. However, the utility of the quantitative research to confirm the outcomes to address in the adapted intervention was less clear, and largely confirmed the apriori hypotheses of our team.

### Comparison to other literature

There are several approaches to develop intervention logic models and core components. We drew on two more mechanistic approaches to clarify the core concepts in our original intervention; Behaviour-Determinant-Intervention logic models (Rolleri, Fuller et al. 2014), and Logic Models of Change (Intervention Mapping) [25]. Although we did find these difficult to apply across our large intervention, we did find that mapping intervention content and gaps was a useful starting point to our process, and crucial to think through how the intervention might be affecting outcomes for individuals.

However, intervention mapping and related, more mechanistic approaches to linking intervention content and effects have been criticised by systems theorists for neglecting the dynamic contexts in which interventions are implemented. Hawe and others have encouraged thinking beyond “ a relatively narrow definition of implementation fidelity conceptualising fidelity to implementation form/content/components versus fidelity to function/theory, recognising that the interventional system is a set of interrelated human and non-human contextual agents and arguably the mechanisms through which the intervention produces change are more important than the actual ingredients [43].”

We did not consciously employ a systems lens for our research; however, we also found that our qualitative research was essential in understanding how some of the content might affect both individual participants, and dynamics within schools which are likely to be important for implementation. These were linked in particular to the active and important role of school administration in secondary schools, and about the potential effects of introducing content framed around challenging gender norms. Challenging gender norms within violence prevention interventions bears consideration, as ‘backlash violence’ has previously described in unintended effects of introducing women’s empowerment interventions to reduce intimate partner violence in contexts where gender norms strictly proscribe women roles [44]. In adolescent school-based interventions, previous studies have also found that interventions with a single-sex focus on girls have led to feelings of exclusion and alienation among boys, and may lead to counter-productive developments and resentment among those who are not direct beneficiaries of interventions [45]. Future iterations of our intervention, the GST, and others, would benefit from a systematic assessment of how the introduction of interventions and their content may affect direct recipients, but also others operating within school systems.

Other adaptation studies base assessments of target behaviours in new populations on practitioner knowledge, focus groups and quantitative data, and compare these findings with the intervention logic model to determine fit [24, 25, 46, 47]. We extended this approach by making full use of quantitative data from our previous trial to conduct a statistical analysis to compare the baseline levels of teacher and peer violence in the population in which the intervention was originally evaluated, with the new population. However, on reflection, this step largely confirmed apriori hypotheses in the team. In our particular case, it may be that the high levels of contextual knowledge precluded the necessity of this step; whereas in other intervention development where less is known about a new target population, this step provides more critical information. Similarly, if the original developers of an intervention are not involved, or are less involved, in an adaptation, further research to understand the new target population and how the intervention may need to be modified may be more important.

## Strengths and limitations

Strengths of the approach described here included a high level of contextual knowledge and program expertise within the Raising Voices team, which provided an apriori sense of what needed to be included in the adaptation. This, combined with a ‘bottom-up’ approach including primary data collection with secondary school students, proved important to triangulate emerging findings. Due to resource constraints, the formative research was conducted in only two purposively selected secondary schools, which we deemed to be broadly representative based on contextual knowledge within the team. However, the formative findings may not generalise outside these schools, and the adaptation may have been strengthened by the inclusion of further schools. Similarly, our quantitative data on secondary schools were collected from the same two schools. Although we had the advantage of quantitative data from 3706 students our previous trial in 42 primary schools to compare to, results should be interpreted with caution. We only carried out formative FGDs with students; carrying out FGDs with teachers and with parents may have provided additional information to inform the adaptation. Although the new content was directed at students, additional information from other stakeholders on acceptability would also be informative for future implementation, particularly from a systems perspective.

### Implications

Our experience of applying existing guidance to adapt a complex violence prevention intervention for primary schools to a secondary school context suggests three main lessons. One, large, complex multicomponent interventions do not lend themselves to mechanistic processes such as intervention mapping. Simpler approaches may provide sufficient clarifications of underlying intervention logic. Two, where there are high levels of contextual knowledge within the intervention adaptation team, extensive research to understand the behaviours or outcomes in the new target population may not necessarily provide any additional insight. Three, qualitative research, especially around understanding the effects of interventions in larger systems, may produce essential insights.

### Next steps

The GST-S has been piloted in two schools and we are currently carrying out a pilot randomised controlled trial of the intervention in a further 8 schools in Kampala and Wakiso districts, Uganda. The aims of this pilot trial are to further refine the GST-S intervention, paying particular attention to ensuring acceptability and understanding, and to understand the feasibility of delivering the intervention in a secondary school context. We will conduct structured observations of intervention activities, half of which will focus on areas identified by Raising Voices to inform refinement. Assuming successful completion of this pilot trial, we aim to secure further funding to carry out a phase 3 trial to assess the effectiveness of the adapted toolkit in secondary schools.

## Conclusions

The systematic, phased approach and practical methods described may be beneficial for application in other adaptation settings. In contexts where there are high levels of contextual knowledge within the team, elucidating core logic models, qualitative research and testing of new content may be the most efficient approach to adaptation.

### Supplementary Information

**Additional file 1: Table S1a.** Description of Measurement Instruments. **Table S1b.** Demographic Characteristics.

**Additional file 2: Table S2.** Summary of Interventions for Dating or Intimate Partner Prevention.

**Additional file 3. **Pretest findings and revisions to the Good School Toolkit.

## Data Availability

The datasets used and analysed during the current study are available from the corresponding author on reasonable request.

## Abbreviations

GST-P: Good School Toolkit for primary schools
GST-S: Good School Toolkit for secondary schools
LSHTM: London School of Hygiene and Tropical Medicine
NGO: Non-Governmental Organisation
