# Supplementary material for: Adapting a complex violence prevention intervention: a case study of the Good School Toolkit in Uganda
Source: BMC Public Health. 2024 Feb 9;24:417. doi: 10.1186/s12889-024-17676-x (PMC10854115; doi:10.1186/s12889-024-17676-x)
Supplement: Supplementary file 1 — Additional file 1: Table S1a. Description of Measurement Instruments. Table S1b. Demographic Characteristics. [file 12889_2024_17676_MOESM1_ESM.docx]

## Additional file 1: Detailed description of the cross-sectional survey measurement instruments

Students' experiences of violence from teachers, peers and others were assessed using the International Study for the Prevention of Child Abuse and Neglect (ICAST) (International Society for the Prevention of Child Abuse and Neglect 2006). We previously used ICAST in the primary schools surveys (Devries, Child et al. 2014). To assess adolescent intimate partner violence, items from various measures were selected, adapted and pretested with adolescents in the Ugandan context, including the WHO Multi Country Study on Women’s Health and Domestic Violence, the UN Multi-country Cross-sectional Study on Men and Violence in Asia and the Pacific, the Conflict in Adolescent Dating Relationships Inventory (CADRI), the Sexual Relationship Power Scale (SRPS) and Ugandan survey measures of exploitive sex.

Table 1 below provides more detail on the constructs, the survey measurement instruments and the adapted question items. Some of the intimate partner measures are designed for adults and only those items relevant to adolescent relationships were selected and amended based on user group and focus group findings and implementer recommendations. All measures or items were pretested in an interview setting with 16 student participants to assess comprehension and acceptable wording for the Ugandan context. Additional survey items were also included to better capture violence from student peers.

**Table S1a. Description of Measurement Instruments**

| **Construct** | **Survey Measure** | **Description of Items** |
| --- | --- | --- |
| Adolescent experience of physical, sexual, emotional violence. | **ICAST** (International Study for the Prevention of Child Abuse and Neglect) as modified for the Good Schools Study (International Society for the Prevention of Child Abuse and Neglect 2006) | Lifetime, past year, and past week measurements of discreet acts of physical, sexual, emotional violence.  Examples include: ‘Cursed, insulted, shouted at or humiliated you’; ‘Slapped you with a hand on your face or head’; ‘Made you take your clothes off when it was not for a medical reason’. |
| Adolescent dating or intimate partner violence | **WHO Multi Country Study on Women’s Health and Domestic Violence**(García-Moreno, Jansen et al. 2005) & **UN Multi-country Cross-sectional Study on Men and Violence in Asia and the Pacific** (Fulu, Jewkes et al. 2013)  **CADRI** (Conflict in Adolescent Dating Relationships Inventory) (Wolfe, Scott et al. 2001, Teitelman, Ratcliffe et al. 2008, Antônio and Hokoda 2009). | Items include exposure to physical, sexual, and emotional violence from partners  Examples include:  Milder forms of aggression more common in adolescent dating relationships (Wolfe, Scott et al. 2001, Teitelman, Ratcliffe et al. 2008, Antônio and Hokoda 2009). |
| Controlling behaviour in adolescent relationships | **Sexual Relationship Power Scale**  (Pulerwitz, Gortmaker et al. 2000) modified for Ugandan adolescent population. | Discreet acts of controlling behaviour by a dating partner.  Examples include: “Most of the time, we do what my partner wants to do” |
| Adolescent transactional sex | **Transactional sex items** selected from various studies and national surveys conducted in Uganda (Neema, Musisi et al. 2004, Darabi, Bankole et al. 2008, Uganda Bureau of Statistics (UBOS) and ICF International Inc. 2012, Choudhry, Ambresin et al. 2015, Wandera, Kwagala et al. 2015) | Items such as: “Do you feel pressured by your partner to do sexual things in return for money, gifts or favours?” |
| Types of adolescent dating and other intimate relationships. | **Types of Adolescent Relationships** items were developed for this study and informed by focus group discussion and advisory user groups. | Items inquire about ‘boyfriend/girlfriend’ relationships and other sexual relationships with a ‘regular partner’ (i.e. not a ‘love’ relationships). |

Table S1b. Demographic Characteristics:

| **Characteristics of Students** | | **Male  (N=260)** | |  | **Female  (N=237)** | |  | **P value** |
| --- | --- | --- | --- | --- | --- | --- | --- | --- |
| N (sex) | | 260 | 52% |  | 237 | 48% |  | n/a |
| **Age (years), mean (SE)** | | 16.5 | (1.11) |  | 15.8 | (1.10) |  | <.001 |
| **Ever worked for money** | | 190 | 73.1% |  | 51 | 21.5% |  | <.001 |
| **Absence from school in previous week (>1day)** | | 40 | 15.4% |  | 51 | 21.5% |  | .07 |
| **Biological mother not alive** | | 23 | 8.9% |  | 15 | 6.4% |  | .29 |
| **Biological father not alive** | | 41 | 15.8% |  | 40 | 17.3% |  | .65 |
| **Has ever had sex** | | 51 | 19.6% |  | 27 | 11.4% |  | .01 |
| **Age at first sex (years), mean (SE)** | | 15.8 | (1.7) |  | 14.8 | (0.53) |  | .69 |
| **Boyfriend/Girlfriend or Regular Partner, lifetime** | | 132 | 51% |  | 136 | 57% |  | .14 |
|  | Ever boyfriend/girlfriend | 124 | 48% |  | 130 | 55% |  | .11 |
|  | Ever regular partner | 38 | 16% |  | 92 | 19% |  | .23 |
| **Exchange of money, gifts, or favours for sex (past year)** | | 52 | 20% |  | 80 | 34% |  | <.001 |
|  | Has felt pressured by partner to do sexual things in return | 10 | 4% |  | 7 | 3% |  | 0.28 |
|  |  |  |  |  |  |  |  |  |
|  |  |  |  |  |  |  |  |  |
