# Supplementary material for: Adapting a complex violence prevention intervention: a case study of the Good School Toolkit in Uganda
Source: BMC Public Health. 2024 Feb 9;24:417. doi: 10.1186/s12889-024-17676-x (PMC10854115; doi:10.1186/s12889-024-17676-x)
Supplement: Supplementary file 2 — Additional file 2: Table S2. Summary of Interventions for Dating or Intimate Partner Prevention. [file 12889_2024_17676_MOESM2_ESM.docx]

## Additional file 2. Review methodology and summary findings.

Literature review of effective, promising or emerging interventions for the prevention of dating (sexual) violence in adolescents.

To inform the development of components to prevent dating violence, we summarised available evidence on what is known about what works to prevent dating violence against adolescents from the international literature. We searched several bodies of literature, including literature on adolescent dating violence, intimate partner violence, violence against young women.

To identify interventions shown to be effective in preventing adolescent dating (sexual) violence, we conducted a review of reviews. The following English language electronic databases were searched for reviews: Medline, PsychINFO, the Cochrane Library, Web of Science and Google Scholar. Only reviews published between January 2000 and July 2015 in a peer-reviewed journal or a book were included. To identify relevant reviews for adolescent dating (sexual) violence, the following search terms were used: ‘prevention’ and ‘sexual abuse' or 'sexual violence' and ‘child’ or ‘children’ or ‘adolescent’ and ‘dating’ or ‘partner’ and ‘evaluation’ or ‘effectiveness’ or ‘efficacy’ and ‘review’.

All relevant primary studies were then extracted from the reviews. For inclusion, the primary studies had to evaluate the effectiveness of a primary prevention intervention for adolescent dating violence, utilise a randomised controlled trial or a controlled trial to measure sexual violence behaviour change outcomes or risk factors. We have included primary studies evaluating different interventions in populations aged 11-18. Although the search for primary studies within reviews was systematic, the full text or detailed abstracts for a small number of relevant studies could not be obtained during the short period available for review and hence the primary studies included here is not exhaustive.

We found only 6 rigorous studies evaluating adolescent dating violence outcomes; of these, 5 interventions were targeting adolescent/youth peer or dating sexual violence in the United States (USA) and one intervention tested in South Africa targeted a combination of HIV and peer/dating sexual violence outcomes. Both the effective and non-effective interventions are included in Table 2 below. Two rigorous studies reporting positive changes in dating violence risk factors were also identified during the review of reviews and the interventions were included for consideration in Table 2.

We also conducted a brief, non-exhaustive search of the broader violence literature to identify strategies which have been successful in the prevention of adult intimate partner violence outcomes or risk factors. Three promising interventions were identified and included for consideration in Table 2.

We concluded that there are several effective programs developed for the USA which have reduced victimisation and/or perpetration of dating violence. There were only two studies identified which evaluated interventions tailored to contexts in middle or low-income countries. There were few rigorous evaluations of dating or intimate violence interventions which target social norms and structures at community and societal levels of the social ecology.

**Table S2. Summary of Interventions for Dating or Intimate Partner Prevention.**

| **Type of violence prevention intervention** | **Effective/**  **promising/**  **emerging intervention.** | **Intervention Names** | **Notes** |
| --- | --- | --- | --- |
| Adolescent dating violence interventions | Effective.  (Evaluated with rigorous designs (RCT or Controlled Trial) and effective for dating violence outcomes.) | - Stepping Stones (South Africa) - Safe Dates (USA) - Coaching Boys to Men (USA) - Shifting Boundaries (School wide/building component) (USA) | Effective interventions all included multiple sessions over several weeks, addressed various issues including healthy relationship skills, gender equity, sexual consent, foster bystander intervention, and some include school-level or community level components. |
|  | Not effective.  (Evaluated with rigorous designs (RCT or Controlled Trial) and not effective for dating violence outcomes.) | - The Acquaintance Rape Prevention Program (USA) - The Men's Project (USA) - Shifting Boundaries (Classroom) (USA) |  |
|  | Promising.  (Evaluated with rigorous designs (RCT or Controlled Trial) and effective for dating violence risk factors.) | - Bringing in the Bystander (USA) - Your Moment of Truth (Kenya). |  |
|  | Emerging. (Interventions which were in development at the time of review, but highly relevant to the context.) | - Skhokho: success for teens. (South Africa). - Power to Girls (Haiti). |  |
| Intimate Partner Violence (Field related to adolescent dating violence.) | Promising, in a related field.  (Evaluated with rigorous designs (RCT or Controlled Trial) and effective for intimate partner violence risk factors.) | - SASA! (Uganda. Reduced the levels of acceptability of violence) - Soul City (South Africa), - Bell Bajao (Ring the Bell) (India) |  |
