# Supplementary material for: Adapting a complex violence prevention intervention: a case study of the Good School Toolkit in Uganda
Source: BMC Public Health. 2024 Feb 9;24:417. doi: 10.1186/s12889-024-17676-x (PMC10854115; doi:10.1186/s12889-024-17676-x)
Supplement: Supplementary file 3 — Additional file 3. Pretest findings and revisions to the Good School Toolkit. [file 12889_2024_17676_MOESM3_ESM.docx]

## Additional file 3. Pretest findings and revisions to the Good School Toolkit.

| **Workshop content to address adaption theme** | **Summary findings from pretesting with students and teachers (including quotes).** | **Revisions to Good Schools Toolkit based on pretest findings** |
| --- | --- | --- |
| **Types of Power**  The workshop ‘Types of Power’ introduces the concept of personal power and both the positive/constructive and abusive application of power in interpersonal relationships. | **Acceptability (Teachers):** High acceptability for positive types of power. Low acceptability from teachers to relinquish their (negative/controlling) ‘power over’ students due to concern for loss of control and legitimacy in schools: *“…to say that, from today onwards your[a teacher’s] power is equivalent with a student...feels like actually…. you are removing it [power over], so he[the teacher] feels he is nowhere to be seen….”(Male Teacher)*  **Acceptability (Students):** Medium acceptability for the workshop suggestion that students be informed that they have ‘power’ as some students could view having ‘power’ as ‘freedom’ and ‘being equal to teachers’, thus disregarding their teachers. Several students sought explicit boundaries to the power afforded to students: *“When you tell students that they have power … they get the concept wrong. They cannot understand it well so, I would suggest that they put a limit to the power the students have. ”* (Male student) | Included a short discussion on the negative effects of abuse of ‘power over’ in a school. Strengthened the idea that ‘power as’ provides teachers with legitimacy and measured authority to discipline students in positive ways.  Maintained workshop on ‘Types of Power’ as training for teachers and student leaders only. Modified the school-wide discussion/activities on ‘power’ for general students population to stress responsibility to use personal power positively and to respect and cooperate with educators as they have ‘power as’. |
| **Gender inequality in school**  The first workshop on ‘Gender in Schools’ is designed to generate empathy and insight as teachers and students listen to the story of a secondary school girl(protagonist) and are asked to think though how they would deal with various gender-related challenges faced by the protagonist. It is follow by a discussion to identify gender imbalances in their own school and how to redress these. The second workshop ‘Challenging Gender Roles’ explores how gender roles are socially constructed and can change over time, and the benefits of gender equality in education. | **Acceptability (Teachers):** Medium acceptability from teachers to provide gender balanced leadership opportunities and academic support. One teacher described support: *“You need to talk to these students to know that … even girls can be leaders.”* (P5, Female teacher) Some teachers reported concerns that gender equitable socialisation at school will erode future patriarchal structures in family life: *“I cannot imagine having a wife in my house that has the same education … giving them[girls] the same opportunities is putting them at a disadvantage after school .”(P1, Male teacher).*  **Acceptability (Students):** Mixed acceptability, with majority of males students not supportive of equal treatment for girls in school, i.e. in terms of leadership, equal gender roles in peer relationships, etc. They are mostly concerned that girls *‘…will assume equal power once they grow up to have families…’* (male student) which will threaten existing patriarchal practices of female submission in marriage. Boys would like to *‘maintain their manhood’* (female teacher) i.e. their masculine identities based on the idea that males are superior. A few male students had dissenting views: *‘…our sisters’ could be of mutual benefit in the future and in the school context.*(male student) Girls internalised examples of gender equality and expressed the intention to have agency in future situations of gender imbalance e.g. in leading their male peers, in resisting pressure for sexual favours etc.  *“My thoughts have changed because previously I was thinking that it is the boy who has the power but now I know that I also have power, … now we know that no one is above the other, we all have to be equal.”(female student)* | Refocused the workshop to not primarily challenge gender roles, e.g. changed name of second workshop from ‘Challenging gender roles’ to ‘Why gender equality in schools?’. Restructured workshop to primarily focus on valuing girls and boys equally, fair treatment for girls and boys, and the mutual benefit for both sexes of providing girls with equal educational opportunities. Participants facilitated to decide which gender roles they would value most.  To prevent alienation of male teachers and students a discussion around ‘gender equity’ vs. ’gender equality’ was included, explaining the importance of maintaining educational support for boys while compensating for girls’ disadvantage and promoting fair treatment. |
| **Peer Violence**  The workshop stimulates discussion and reflection on the types and causes of peer violence. The workshop also facilitates an activity were participants reflect on the skills they can use to respond to different scenarios of peer violence. | **Acceptability (Teachers):** High acceptability for workshop as teachers related strongly to workshop content i.e. teachers contributed many personal examples of past peer violence which created empathy for their students experiencing the same. (Observations: Teacher Workshop; FGD Teachers). Teachers expressed the willingness to put an ‘*end to [peer] violence’* through policy, allowing student to report incidents and following up. Some teachers were concerned that most school teachers are employed part-time and deal with more than 40 to 60 students per class, limiting their ability to attend to peer violence.  **Acceptability (Students):** High acceptability as students could relate to their experience of peer violence to the workshop content. Students were also accepting of suggestions to stop peer violence, for example a male student noted that *‘working together as a team can easily lead to violence prevention.’* One student also explained: “ *What I learnt is that you have to think first, if I do something wrong to this person and the same thing happened to me, how would I feel…” .(*Female student) Students reported that often *‘the teachers’ failure to take action promotes it[peer violence.”* (Male student) | The Good School Toolkit is designed to support development of a policy on peer violence and disciplinary action that will follow. This should support part-time teachers with implementation.  Discussion point added: (Note to teachers) Peer violence policies, teacher who are responsive to student reports and peer-led ‘students courts’ are some of the measures a school can help to deter peer violence. |
| **Shared Human Rights**  Workshop simulates a game which creates an immediate experience of one group having power over another to disregards their rights as well as the role of duty bearers to protect rights. | **Acceptability (Teachers):** Teachers participated enthusiastically in the game during the teachers’ workshop but the discussion showed mixed acceptability to present the workshop content to students. Some teachers may resist informing students of their human rights as it may lead to a shift in the power dynamics. *“...they will think their power is being taken away...they will think the children are going to be unruly, and they are not going to be easily governed in the school...they are not going to be easily controlled.”* The majority of teachers requested that students’ responsibility be introduced alongside student rights.  **Acceptability (Students):** Students participate enthusiastically in the workshop and could comprehend all content, but noted concerns that open discussion of shared rights may cause some students to *“… abuse teachers and not listen to what teachers say…”(*male student) and to *“undermine the teachers claiming that they have equal rights*. (male student)”. Students also recommended that *“…they should teach students their rights and then caution them.”* (male student) | Discussion point added: (Note to teachers) Students sometimes misbehave or do not fulfil the responsibilities which accompany certain rights. Positive discipline methods provide an alternative to maintaining school discipline while not disregarding students’ rights.  Workshop discussion point added: (Note to students) With shared rights come the responsibility to respect the rights of others, i.e. to afford others their right to respect, physical safety, etc. With shared rights also come responsibility to make the most of access to an education, to not damage the school facilities, etc. |
| **Peer and Dating Relationships**  Workshop contrasts ‘helpful’ and ‘hurtful’ peer relationships. Hurtful relationships lead to violence. Steps students can take to avoid hurtful peer relationships are discussed. | **Acceptability (Teachers):** Most of the teachers accepted the workshop content related to friendships and general peer relationships. Mixed acceptability from teachers for discussing dating relationships with students. Most schools prohibit students from having dating relationships at school due to the risk of pregnancy. Some teachers were concerned that addressing dating relationships would promote sexual relationships while other teachers support open discussion: *‘…sexual relationships are going on in our schools... we have also seen them [teachers] with students. So it is very ok to talk about it, and to create that awareness …”*  **Acceptability (Students):** High acceptance from male and female students. | Refocused the session to address friendships and other peer relationships. The single discussion point which prompts teachers to apply this session to dating relationships was made optional. |
|  | **High Acceptability**  **Medium Acceptability**  **Low Acceptability**  **Mixed Acceptability** |  |
